# A User-Centered Interface Design Framework for the DELONELINESS System in Older Adults: Design Indicator Development and Prioritization

**DOI:** 10.2196/88263

**Published:** 2026-03-06

**Authors:** Yi Zhou, Jessica Rees, Faith Matcham, Michela Antonelli, Sebastien Ourselin, Wei Liu

**Affiliations:** 1 Department of Engineering, King's College London, Strand Campus, Strand, London, WC2R 2LS, United Kingdom, 44 020 7836 5454; 2 Department of Global Health and Social Medicine, King's College London, London, United Kingdom; 3 School of Psychology, University of Sussex, Brighton, United Kingdom; 4 School of Biomedical Engineering & Imaging Sciences, King's College London, London, United Kingdom

**Keywords:** older adults, loneliness, digital health, user-centered design, human-computer interaction, interface design

## Abstract

**Background:**

Loneliness among older adults has become a major public health concern associated with cognitive decline, depression, and increased health care use. The advancement of digital health technologies such as wearable devices, smart home systems, and mobile health apps provides new opportunities to monitor and mitigate loneliness through continuous physiological and behavioral assessment. However, the effectiveness of such technologies largely depends on user interface design, ensuring that older adults can understand, trust, and comfortably engage with the technology. Existing research on interactive platforms and user interfaces for psychological and emotional monitoring has mainly focused on usability testing and technology feasibility, with limited attention to structured design frameworks that integrate psychological, emotional, and accessibility dimensions for older adults. Furthermore, most studies rely on qualitative assessments and lack quantitative prioritization of design indicators.

**Objective:**

This study aimed to build on the DELONELINESS (Design for Healthy Ageing: a Smart System to Decrease Loneliness for Older People) system to develop a user-centered hierarchical framework of interface design indicators for older adults.

**Methods:**

A mixed methods design was applied, integrating literature search, qualitative focus group analysis, and expert consultation to build an initial indicator pool. A hierarchical indicator structure with 7 first-level and 26 second-level indicators was developed. The analytic hierarchy process was used to assign indicator weights through surveys of 20 experts with academic or professional experience in human-computer interaction, digital health, gerontology, and health informatics. Based on the weighted results, 3 interface design solutions were developed and comparatively evaluated using the Technique for Order Preference by Similarity to Ideal Solution.

**Results:**

All expert judgment matrices satisfied the analytic hierarchy process consistency requirement (consistency ratio <0.1). The Kendall coefficient of concordance indicated good agreement among experts for both first-level indicators (W=0.313; *P*<.001) and second-level indicators (W=0.156; *P*<.001). Among the 7 first-level indicators, trust and safety (weight=0.206), ease of use (weight=0.187), and accessibility (weight=0.167) received the highest weights, indicating their importance in enhancing user confidence and engagement. Technique for Order Preference by Similarity to Ideal Solution evaluation results showed that design solution 2 achieved the highest overall performance score (relative closeness coefficient C=0.877), emphasizing clear interaction pathways, visual clarity, and guided feedback as key factors for optimal usability.

**Conclusions:**

This study developed a user-centered framework for interface design in loneliness monitoring among older adults by integrating user insights, literature-derived indicators, and expert consensus, and providing a structured data-driven approach to prioritizing design requirements. The proposed framework bridges subjective user experience with objective evaluation, offering practical guidance for developing empathetic, inclusive, and trustworthy digital mental health technologies for older adults.

## Introduction

With the rapid growth of the global aging population, loneliness has become a major public health concern in aging societies. It is estimated that up to 1 in 4 older adults worldwide currently experience social isolation [1-3]. Research has demonstrated that older adults are particularly vulnerable to loneliness and social isolation. As people age, they may experience loneliness and social isolation due to reduced social interactions caused by various factors, including physical and cognitive changes and losses, living alone, or the loss of family or friends [4-6]. Prolonged loneliness or social isolation has been linked to a higher risk of depression, dementia, physical decline, chronic illness, and even premature mortality [7,8]. Furthermore, loneliness increases the demand for health care and long-term support services, placing substantial strain on health care systems [5]. Therefore, there is an urgent need for cost-effective and accessible interventions to alleviate loneliness among older adults.

In recent years, the development of digital health technologies such as wearable devices, smart home systems, and mobile health apps has created new opportunities to monitor and mitigate loneliness through continuous physiological and behavioral assessment. For example, smartwatches and sensor-based wearables can monitor posture as well as physiological parameters linked to loneliness, such as heart rate, respiration, and blood oxygen levels [9-12]. Microphone or camera-based smart furniture and environmental monitoring devices can detect activity patterns, and artificial intelligence (AI)-driven interactive platforms are increasingly being used to provide emotional support and facilitate social interaction among older adults [13-15]. Advances in flexible electronics and smart textiles have further enabled the seamless integration of monitoring technologies into garments or furniture, creating unobtrusive and continuous passive monitoring solutions [10,16,17]. These systems can capture multimodal signals such as heart rate, respiration, and movement to infer emotional and social states and deliver personalized interventions. However, despite significant progress in data collection and AI-based analytical technologies, the effectiveness of these systems largely depends on the design of their user interfaces [18,19]. The design of user interfaces, particularly those for older adults, should ensure that users can understand, trust, and comfortably interact with the system, while also promoting inclusivity and long-term engagement to support psychological well-being [20].

Older adults may face greater challenges when interacting with digital technologies and user interfaces due to limited digital literacy, cognitive and sensory changes, and age-related physical decline [21]. For instance, the loss of fine motor skills can make it difficult to interact with small virtual buttons [22]. In addition, visual impairments can hinder the perception of detail, color, and contrast, and hearing loss can reduce responsiveness to auditory feedback cues [23]. Therefore, the design of interactive platforms and user interfaces should consider these age-related changes to ensure inclusivity and usability. Beyond these physiological and cognitive considerations, emotional comfort and psychological acceptance are essential in determining older adults’ engagement with digital systems. Many older adults may experience stigma associated with mental health or hold generational beliefs that discourage emotional disclosure [24,25]. As a result, they may be reluctant to use mental health-related technologies, perceiving them as intrusive or impersonal. Increasing evidence suggests that systems lacking empathy, warmth, and reassurance may unintentionally reinforce feelings of loneliness rather than alleviate them [26,27].

Although digital mental health interventions have gained increasing attention, research specifically focusing on the design of interactive platforms and user interfaces for psychological and emotional monitoring among older adults remains limited [20]. Most current studies have mainly focused on usability testing or technical feasibility, such as investigating conversational agents, social platforms, and mobile apps for emotional well-being in older adults [28-31]. And these studies mainly adopted in-depth qualitative evaluation or user testing, providing valuable insights into user experiences and contextual needs. However, they generally do not extend to systematic or quantitatively prioritized design frameworks that integrate technological, emotional, and psychological factors. To address this gap, this study developed a user-centered framework for interface design in loneliness monitoring technologies for older adults. By integrating qualitative focus group analysis, systematic literature search, and expert consultation, this study established a comprehensive design indicator framework based on the analytic hierarchy process (AHP) [32]. Interface design solutions were then developed according to these indicators and evaluated using the Technique for Order Preference by Similarity to Ideal Solution (TOPSIS) [33]. Building on the DELONELINESS (Design for Healthy Ageing: a Smart System to Decrease Loneliness for Older People) smart textile loneliness monitoring system we previously developed [34], we aimed to construct a hierarchical design indicator model reflecting the cognitive, emotional, and functional needs of older adults. Expert consensus was used to assign weights to these indicators, followed by the development and evaluation of interface design solutions. Through this process, the study generates a quantified prioritization of interface design requirements for loneliness monitoring in older adults and provides an evidence-based decision model that can be adapted to other age-friendly digital mental health interfaces. This integrated approach bridges user-centered design principles with data-driven decision analysis, providing a solid foundation for the future development of digital mental health technologies and interactive platforms for older adults.

## Methods

### Study Design

This study applied a mixed method to prioritize interface design indicators and to develop interface solutions for the DELONELINESS system for older adults. The DELONELINESS system is a smart garment and furniture-based platform designed to monitor loneliness in older adults [34]. Through its associated user interfaces, the system provides older users with real-time feedback on various physiological and behavioral parameters such as heart rate, respiration rate, and daily activity levels, while also visualizing the current loneliness status and offering recommendations for health-promoting activities.

**Figure 1.**
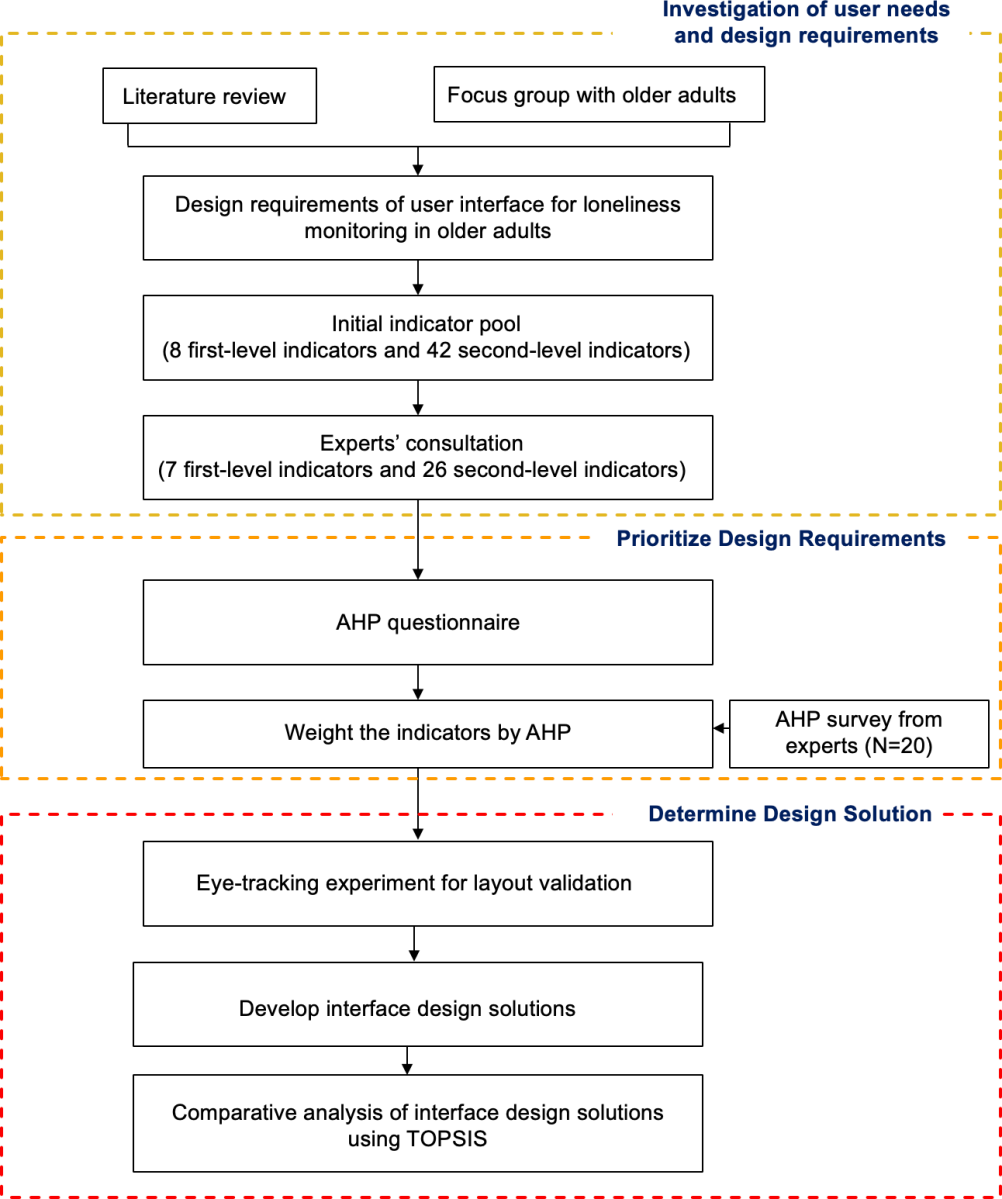
The research process. AHP: analytic hierarchy process; TOPSIS: Technique for Order Preference by Similarity to Ideal Solution.

An initial pool of design indicators was constructed based on a literature review and qualitative analysis of previous focus groups with older adults [34]. Following screening by experts of human-computer interaction (HCI), an AHP survey was conducted to determine the relative weights and priorities of the indicators. To further verify the applicability of the design indicators derived from the AHP analysis in actual interface structures, an eye-tracking experiment was conducted to compare different interface layouts in terms of information accessibility and cognitive clarity. Then, the findings of the eye-tracking experiment and user requirements with higher weight values derived from AHP were integrated to produce three interface design solutions for comparative analysis. Finally, the design solutions were evaluated using the TOPSIS to validate their overall feasibility. The research process is presented in Figure 1.

### Construction of Initial Indicator Pool

The initial indicator pool was constructed based on a comprehensive literature search and qualitative analysis of focus groups with older adults. The literature search was conducted across 4 major databases including IEEE Xplore, ACM Digital Library, Scopus, and Web of Science to identify studies related to older adults, health technology platforms, and interface design. The search string was defined as:

(“older adults” OR “aging people” OR “aging” OR “senior citizens” OR “elderly”) AND (“interface design” OR “user interface” OR “interaction design” OR “human computer interaction” OR “user experience”) AND (“loneliness monitoring” OR “mental health” OR “psychological wellbeing” OR “digital health”)

**Figure 2.**
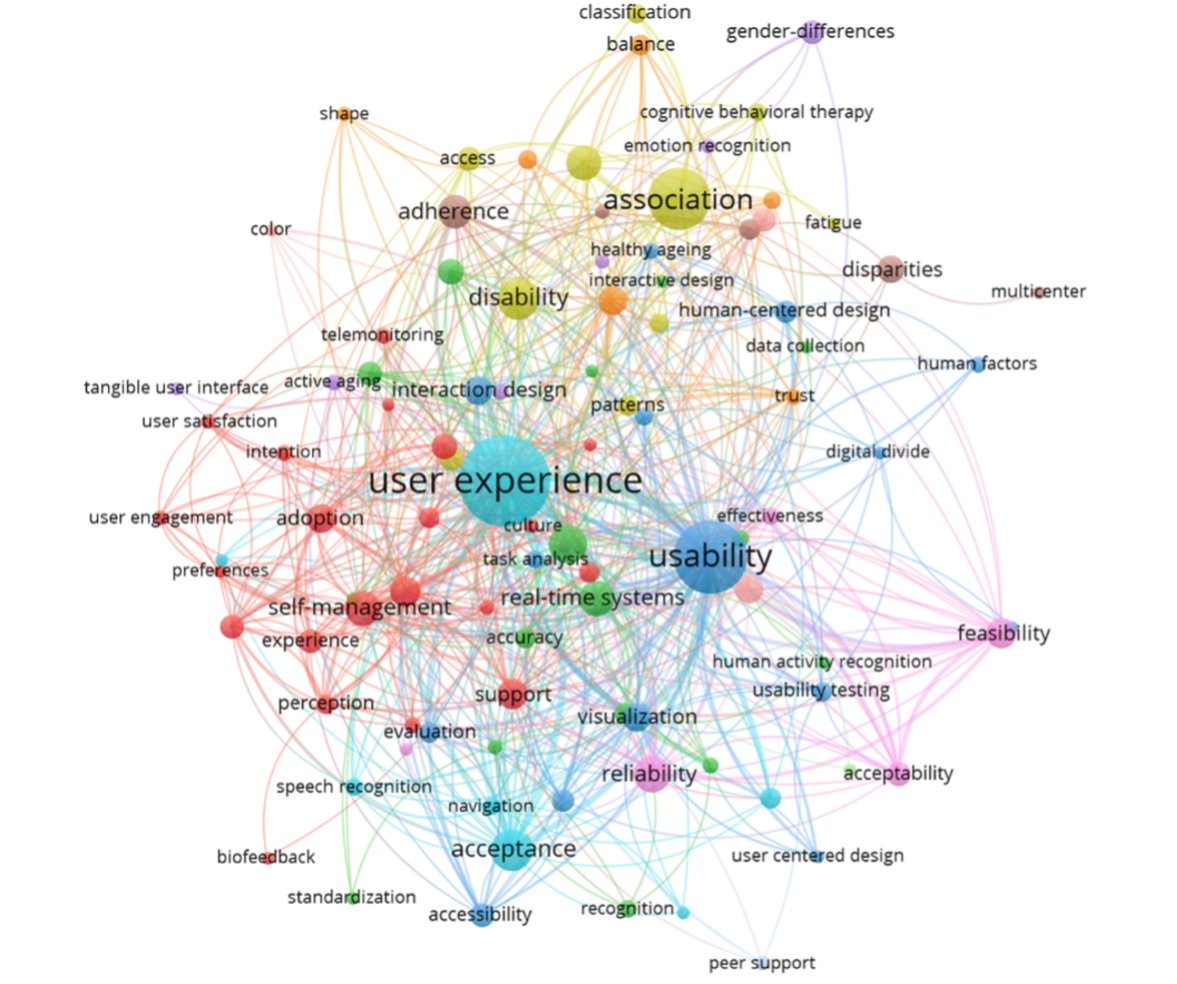
Co-occurrence analysis of high-frequency terms in research of digital health and interface design for older adults.

From the search results, 3522 highly relevant papers with more than 10 citations were selected for further analysis. First, nondescriptive and generic terms (such as “health” and “technology”), as well as duplicates were removed. Additionally, high-frequency terms were extracted using term frequency–inverse document frequency analysis and visualized [35]. As shown in Figure 2, the node size and color represent word frequency, with larger nodes indicating higher frequency, while node color represents keyword clustering. From this analysis, high-frequency terms were synthesized into preliminary interface-related indicators such as usability, feasibility, accessibility, and reliability.

Additionally, qualitative thematic analysis was conducted on the results of our previous focus groups with older adults [34]. These focus groups explored older users’ lived experiences of loneliness, expectations, and concerns regarding digital health and monitoring technologies for loneliness across diverse living contexts. The transcripts were coded using open coding. Two researchers independently coded data and resolved discrepancies through discussion to ensure consistency. Statements and discussion related to comprehensibility, trust and safety, emotional comfort, and personalization of user interfaces were coded (Multimedia Appendix 1). These findings provided a user-centered perspective and complemented the design requirements identified through the literature search.

By integrating results from both the literature and focus group analysis, an initial indicator pool comprising 8 first-level and 42 second-level indicators was established to demonstrate different aspects of user interface design for loneliness monitoring in older adults. Then, consultation was undertaken with 8 experts in HCI design. These experts were not involved in the AHP survey. They reviewed the combined results of the literature review and focus group analysis, deleted redundant or overlapping items, and suggested additional indicators where appropriate. Finally, a refined hierarchical indicator structure containing 7 first-level indicators and 26 second-level indicators was determined.

### Inclusion Criteria of Experts

To evaluate and prioritize the interface design indicators, experts were recruited to participate in the AHP survey. To ensure the validity and reliability of the weighting results, the inclusion criteria for experts were as follows: (1) professional background: experts were required to have academic or professional expertise in HCI, digital health, gerontology, or health informatics; (2) experience: at least 5 years of professional experience in the relevant fields; (3) academic or professional rank: an intermediate level or higher professional title; and (4) voluntary participation: willingness to participate in the survey and agreement of informed consent for the use of their responses in the research.

A total of 23 experts meeting the above criteria were invited to participate. 20 out of 23 (86.96%) experts completed the questionnaire. All these experts held a master’s degree or higher and were mainly composed of university lecturers, postdoctoral researchers, clinical experts, and industry professionals with work experience in HCI design and digital health. The demographic characteristics of the experts are summarized in Table 1.

**Table 1.** Characteristics of the analytic hierarchy process (AHP) participants (N=20).

Characteristics	Values, n (%)
Professional background	
Human-computer interaction	7 (35)
Digital health	6 (30)
Gerontology	4 (20)
Health informatics	3 (15)
Occupation	
University lecturers	5 (25)
Postdoctoral researchers	3 (15)
Clinical experts	4 (20)
Industry professionals	8 (40)
Seniority (y)	
6‐10	14 (70)
11‐15	3 (15)
16‐20	2 (10)
20	1 (5)
Professional title	
Middle	5 (25)
Associate senior	8 (40)
Senior	7 (35)
Education	
Master’s degree	8 (40)
Doctoral degree	12 (60)

### Weight Assignment of Indicators

Based on the refined indicator pool, we developed an AHP questionnaire (Multimedia Appendix 2) comprising 7 first-level indicators and 26 second-level indicators. The questionnaire was structured as a pairwise comparison matrix, requiring experts to judge the relative importance of each indicator in relation to the others. To guide the evaluation, we adopted the Saaty 1 to 9 scale, which is widely applied in multicriteria decision analysis [32]. On this scale, a score of “1” represents equal importance, while larger odd numbers indicate greater importance, such as “3” (moderate importance), “5” (strong importance), “7” (very strong importance), and “9” (absolute importance). Even numbers (2, 4, 6, and 8) were used to allow for more nuanced judgments between categories. This structured approach ensured that expert evaluations could be translated into quantitative weights for each indicator [36,37]. The survey was distributed via email to 23 eligible experts, and 20 experts completed the questionnaire.

The collected data were first entered into Microsoft Excel and then imported into SPSS software (version 29.0.2.0; IBM Corp) to calculate the weights of both first-level and second-level indicators. For each judgment matrix, the largest eigenvalue and its corresponding eigenvector were calculated to determine the relative weights of indicators. The consistency ratio (CR) was used to verify the logical consistency of expert judgments, with CR <0.1 indicating acceptable consistency. To further assess the degree of agreement among experts, the Kendall coefficient of concordance (Kendall W) was applied, with values ranging from 0 to 1, where higher values represent greater agreement [38]. Results were considered statistically significant when *P*<.05. Finally, the resulting pairwise comparison matrix from all experts was integrated to obtain the final weight of each indicator. The weighted values were normalized so that the sum of all indicator weights equaled 1. These results provided a prioritized structure of interface design requirements, informing the design of following user interface solutions.

### Development of Design Solutions

After the weight assignment of design indicators through AHP, we examined the applicability of the prioritized indicators to interface layouts. At this early prototyping stage, an eye-tracking experiment was conducted to inform layout selection and structural design decisions rather than to evaluate end-user experience. The eye-tracking study was designed to assess how different layout types (list-detail, grid, and feed layouts) influenced information localization and visual-cognitive efficiency within the DELONELINESS application. A Tobii Fusion eye tracker (60 Hz) was used to record gaze behavior. The participants were the same 20 experts who had taken part in the previous AHP evaluation. Each expert completed information search tasks in various layout types through locating specific interface elements and triggering screen transitions by clicking on the target area. The system automatically recorded each participant’s gaze trajectory and task completion time.

We analyzed 2 primary performance metrics, including gaze path distribution and task completion time (TCT). Based on the results, 3 user interface design solutions were developed. These design solutions varied in layout and user experience but were all aligned with the prioritized requirements identified in the weight assignment stage.

### Evaluation of Design Solutions

To evaluate the feasibility of these 3 design solutions, the same group of experts from the AHP phase was invited to participate in a structured assessment. Experts rated each design solution against the indicators obtained from the previous AHP analysis. Ratings were collected using a 9-point Likert scale, ranging from 1 (“Strongly Disagree”) to 9 (“Strongly Agree”) to quantify the performance of each design solution across the 7 first-level and 26 second-level indicators. After collecting the ratings, the highest and lowest values were excluded, and the mean of the remaining scores was calculated as the composite score for each solution.

To evaluate the reliability of expert ratings, the intraclass correlation coefficient (ICC) was computed. A 2-way random mixed model with absolute agreement was applied. ICC values greater than 0.60 were considered indicative of good consistency, while values above 0.75 indicated excellent consistency. Additionally, we applied the TOPSIS to calculate the evaluation score for each design solution. This method constructs an ideal solution (the best performance across all indicators) and a negative ideal solution (the worst performance across all indicators) and measures the relative distance of each design alternative to these 2 reference points [33]. By calculating the closeness coefficient, these 3 solutions were ranked and the most suitable interface design for the DELONELINESS system was identified.

### Ethical Considerations

This research received ethical approval from the Research Ethics Committee of King’s College London (reference: LRS/DP-24/25‐34602). Prior to participation, all participants received a participant information sheet and a consent form via email, which outlined the study objectives, participants’ rights, and the voluntary nature of participation. Electronic informed consent was obtained before data collection. Files containing participants’ names and contact details were stored separately from research data in password-protected documents, accessible only to the core research team. No compensation was provided for participation.

## Results

### Weight Assignment of Indicators

To evaluate the reliability of expert judgments, a consistency test was performed on each expert’s pairwise comparison matrix. The CR of all matrices was less than 0.1, indicating that all expert judgments met the consistency requirement. In addition, to assess the overall agreement among experts in judging the importance of indicators at each hierarchical level, the Kendall W was calculated for both first and second-level indicators. The Kendall’s W value was 0.313 for the first-level indicators (*χ*²_₆_=37.606, *P*<.001) and 0.156 for the second-level indicators (*χ*²_₁₉_=59.286, *P*<.001). These results demonstrate a satisfactory level of logical consistency among experts, supporting the validity of further weight calculation and coordination analysis.

After the consistency test, the AHP was applied to calculate the normalized weights and combination weights of each indicator. The detailed weighting results are presented in Multimedia Appendix 3. Among the first-level indicators, trust and safety (0.206) had the highest overall weight, followed by ease of use (0.187) and accessibility (0.167), indicating that these dimensions were considered the most critical factors in the interface design of the DELONELINESS system. In contrast, emotional comfort (0.080) and personalization (0.092) had relatively lower weights. At the second level, the highest-weighted indicators included user ability to modify permissions (0.084), clear interaction pathways (0.667), and transparent data collection (0.566), showing that user autonomy and system transparency were regarded as key determinants of older user trust and engagement. Other highly ranked indicators, such as offline usability under poor network conditions (0.550) and clear interaction areas (0.521), highlight the importance of system robustness and interface clarity for older adult users. Overall, these findings indicate that experts prioritized factors that enhance trust, safety, and operational simplicity over purely aesthetic or emotional aspects. The resulting hierarchical weighting structure provided a quantitative foundation for the further design and evaluation of user interface solutions.

### Development of Design Solutions

In the interface design of the DELONELINESS system, we first examined how different layout structures influenced visual-cognitive efficiency and interaction performance. A one-way ANOVA was conducted to analyze the eye-tracking data across different interface layout types. The results revealed significant differences in TCT (*P*<.05), indicating that interface structure had a significant impact on visual localization efficiency and interaction performance.

Among the 3 layouts, the list–detail layout performed best on TCT (mean 1.33, SD 0.33), lower than the grid layout (mean 1.37, SD 0.33) and the feed layout (mean 1.76, SD 0.65). These findings suggest that the list-detail layout more effectively guided visual attention, reduced information retrieval load, and improved task execution efficiency.

**Figure 3.**
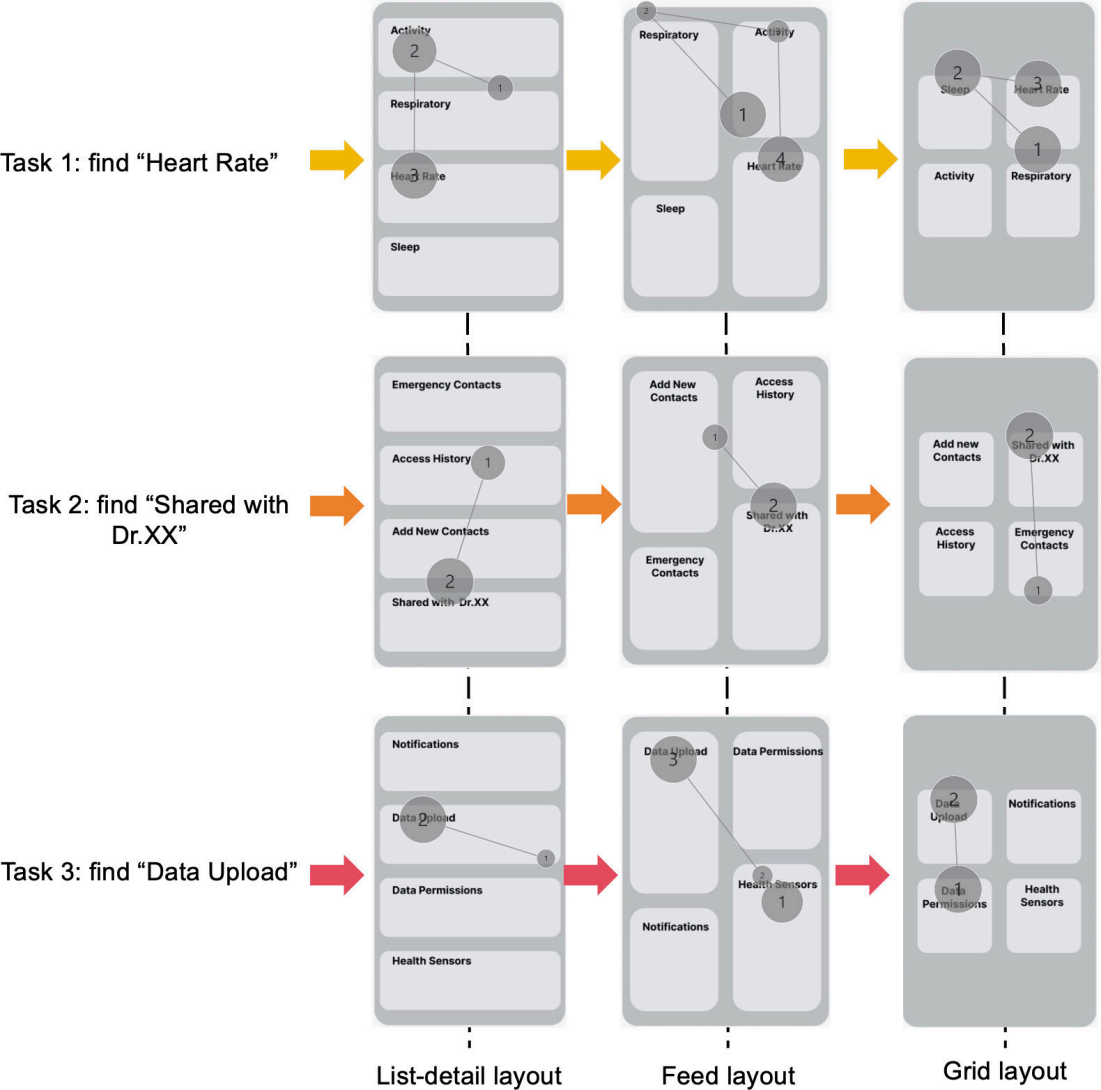
Gaze paths across 3 interface layout types in different tasks.

As shown in Figure 3, the list-detail layout showed a more focused and linear visual scanning path, with fixation points concentrated in core information areas. In contrast, the grid layout produced multiple scattered fixation hotspots, whereas the feed layout presented more diffuse but continuous scanning patterns. Overall, the list-detail layout demonstrated outstanding visual and operational efficiency, providing evidence for the following interface prototype design.

**Figure 4.**
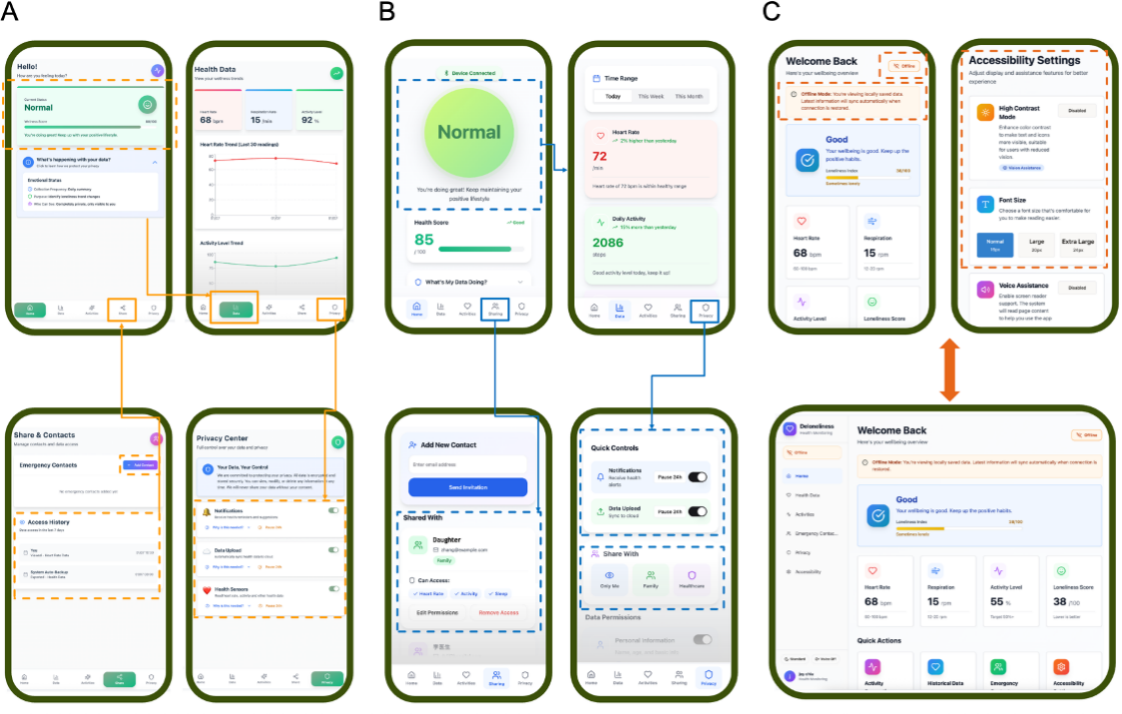
User interface design solutions for DELONELINESS (Design for Healthy Ageing: a Smart System to Decrease Loneliness for Older People) system: (A) design solution 1, (B) design solution 2, and (C) design solution 3.

Based on these findings, the hierarchical weighting structure derived from the AHP was integrated into the interface design process. Among all design indicators, trust and safety, ease of use, and accessibility were identified as the most important dimensions. Therefore, 3 interface design solutions were developed (Figure 4). Each design followed the same hierarchical weighting framework and maintained a consistent functional architecture, including the home, data, activities, sharing, and privacy modules. However, they differed in the expression and emphasis of high-priority dimensions.

As shown in Figure 4, design solution 1 prioritizes trust and safety and comprehensibility by providing users with transparent control over their data and privacy settings. The homepage displays a simplified loneliness status section, using soft colors and positively framed language to convey feedback. Below, the section titled “What’s Happening With Your Data?” allows users to quickly access information about ongoing data collection and its purposes. The “Share and Contacts” page visualizes access permissions and emergency contacts and enables users to view their data-sharing history, which enhances both their sense of security and control. Within the “Privacy Center,” users can manage notifications and data permissions and pause data uploads at any time.

Design solution 2 (Figure 4) focuses on ease of use and comprehensibility, addressing users’ needs for clarity and intuitive interaction. The homepage features a large central status indicator, accompanied by a simplified health score and encouraging feedback. Navigation follows a linear guided structure to reduce users’ cognitive load. Users can access “Quick Controls” for notifications and data uploads, while the “Privacy” section integrates all permission settings in an intuitive toggle-based layout. Additional cues such as tooltips and contextual guidance can help older adults navigate unfamiliar interactions. This design emphasizes clear interaction pathways and immediate guidance, enhancing operational ease and user confidence during daily use.

Design solution 3 (Figure 4) emphasizes accessibility and ensures usability under unstable network conditions. The interface adapts seamlessly across smartphones and tablets and supports a low-bandwidth mode to enable offline access. Key health and loneliness indicators can remain available locally and synchronize automatically once connectivity is restored. The “Accessibility” section offers high-contrast modes, enlarged fonts, and voice-assist features, improving inclusivity and reliability. This design ensures that the system remains usable for older adults with sensory limitations or those living in poor network environments.

### Evaluation of Design Solutions

To evaluate the feasibility of these three design solutions, the TOPSIS was applied to compare their performance and identify the optimal interface design for the DELONELINESS system. First, experts rated each design solution across 7 first-level and 26 second-level indicators using a 9-point Likert scale. To assess the reliability of expert ratings, the ICC was calculated. The ICC values for design solution 1, design solution 2, and design solution 3 were 0.761, 0.653, and 0.747, respectively. All ICC values exceeded 0.6, indicating moderate to good consistency among experts. The mean scores of each design solution are shown in Table 2. At the first indicator level, design solution 2 achieved the highest mean scores across most dimensions, particularly in comprehensibility (6.39), ease of use (6.50), and feedback and support (5.67), demonstrating its guided and user-friendly interaction design. Design solution 1 emphasized trust and data transparency and achieved the highest score in trust and safety (6.17). Design solution 3 focused on accessibility and obtained the relatively higher scores in accessibility (5.23) and comprehensibility (6.22). At the second indicator level, design solution 2 achieved higher average scores in clarity of visual elements (6.61), consistency of interface layout (6.33), and clear interaction areas (6.28), suggesting experts’ preference for its clear visual hierarchy and intuitive structure. Design solution 3 performed well in cross-device access (5.43), multisystem compatibility (5.33), and accessibility support functions (4.61), indicating good robustness in supporting diverse usage contexts. Although design solution 1 received slightly lower overall scores, it performed best in transparent data collection (6.17) and user authorization of functions (6.06), demonstrating perceptions of trust and control.

**Table 2.** Mean expert ratings of the 3 interface design solutions.

	Design solution 1	Design solution 2	Design solution 3
First-level indicators, mean (SD)			
Comprehensibility	5.28 (0.82)	6.39 (1.14)	6.22 (1.07)
Ease of use	5.26 (1.05)	6.50 (0.64)	6.06 (1.02)
Trust and safety	6.17 (0.98)	5.61 (1.32)	6.11 (1.00)
Feedback and support	5.06 (1.00)	5.67 (1.27)	5.39 (0.82)
Emotional comfort	4.22 (0.72)	5.33 (1.17)	5.22 (1.05)
Personalization	4.22 (1.02)	5.11 (1.15)	5.06 (0.92)
Accessibility	4.44 (0.70)	4.78 (1.23)	5.23 (1.09)
Second-level indicators, mean (SD)			
1.1 Consistency of interface layout	5.17 (1.19)	6.33 (1.45)	6.22 (0.91)
1.2 Clarity of visual elements	5.11 (1.06)	6.61 (1.67)	6.28 (1.08)
1.3 Simplicity of information content	4.94 (1.14)	6.22 (1.38)	6.39 (0.97)
2.1 Clear interaction areas	5.06 (1.08)	6.28 (1.76)	6.06 (1.15)
2.2 Support for multimodal interaction	5.28 (1.16)	6.33 (1.62)	5.83 (0.87)
2.3 Easy correction of errors	5.28 (0.98)	6.22 (1.72)	6.28 (1.02)
2.4 Clear interaction pathways	5.17 (1.14)	6.00 (1.34)	6.06 (1.19)
3.1 Transparent data collection	6.17 (0.94)	6.11 (0.91)	5.72 (1.29)
3.2 User authorization of functions	6.06 (1.15)	6.00 (1.02)	5.96 (1.22)
3.3 User ability to modify permissions	5.39 (0.92)	5.72 (1.21)	5.67 (1.03)
4.1 Real-time health status feedback	4.78 (1.16)	5.50 (1.28)	5.56 (1.19)
4.2 Immediate guidance	4.56 (1.14)	5.22 (1.54)	5.61 (1.33)
4.3 Online assistance	4.50 (1.32)	5.33 (1.44)	5.39 (0.94)
5.1 Emotionally supportive language	4.11 (0.97)	5.06 (1.46)	5.11 (1.04)
5.2 Emotionally supportive images	4.39 (0.94)	5.28 (1.52)	5.28 (1.12)
5.3 Integration with daily life scenarios	4.50 (1.06)	5.28 (1.27)	5.00 (1.21)
6.1 Customizable fonts	4.06 (1.23)	4.89 (1.41)	4.94 (1.10)
6.2 Customizable language	4.39 (1.05)	4.72 (1.59)	4.89 (1.02)
6.3 Customizable notifications	4.00 (1.05)	4.78 (1.35)	4.83 (1.27)
6.4 Customizable mode	4.39 (0.98)	5.00 (1.52)	5.06 (1.34)
6.5 Customizable mood input	4.28 (1.17)	4.89 (1.63)	4.83 (1.06)
7.1 Cross-device access	4.33 (0.91)	4.61 (1.38)	5.43 (1.04)
7.2 Multisystem compatibility	4.28 (0.89)	4.72 (1.59)	5.33 (1.15)
7.3 Accessibility support functions	4.17 (0.76)	4.44 (1.41)	4.61 (1.29)
7.4 Offline usability under poor network conditions	4.22 (0.78)	4.78 (1.61)	4.98 (1.38)

Based on the expert ratings and the AHP indicator weights, the TOPSIS method was used to evaluate the performance of the 3 design solutions. The distances to the positive and negative ideal solutions and the overall closeness coefficients are presented in Table 3. The results showed that design solution 2 had the highest overall score, followed by design solution 3 and design solution 1. These findings indicate that design solution 2 was closest to the ideal interface configuration, which can offer the best balance of trust, usability, and accessibility.

**Table 3.** Technique for Order Preference by Similarity to Ideal Solution (TOPSIS) evaluation of design solutions.

	Distance to positive ideal solution (D^+^)	Distance to negative ideal solution (D^–^)	Relative closeness (C)	Ranking
Design solution 1	0.999	0.002	0.002	3
Design solution 2	0.132	0.942	0.877	1
Design solution 3	0.318	0.855	0.728	2

## Discussion

### Principal Findings

This study aimed to construct a user-interface design indicator model for the DELONELINESS system, a smart textile system for monitoring loneliness among older adults. To comprehensively integrate design requirements for health technology platforms targeting older users, we applied a mixed methods approach, combining a systematic literature search, qualitative focus group analysis, and expert consultation. These processes led to the construction of a design indicator framework, consisting of 7 first-level indicators and 26 second-level indicators. The AHP was then applied to assign relative weights to each indicator. Based on the derived weights, 3 interface design solutions were developed and evaluated by experts using the TOPSIS to identify the optimal configuration. The findings indicate that trust and safety, ease of use, and accessibility were prioritized over other design dimensions, highlighting the central role of transparency, cognitive simplicity, and inclusive access in interface design for monitoring loneliness among older adults.

The AHP results constructed a hierarchical weighting structure. Among the 7 first-level indicators, trust and safety (0.206), ease of use (0.187), and accessibility (0.167) were identified as the most important dimensions, indicating that data transparency, simplified user operations, and accessibility under different physical and technological conditions are essential for enhancing older adults’ confidence and engagement with digital systems. At the second level, user ability to modify permissions (0.084), clear interaction pathways (0.067), and transparent data collection (0.057) received the highest weights, demonstrating the key role of user autonomy and system transparency in fostering trust and usability.

Based on these priorities, three interface design solutions were developed. Each solution followed the same hierarchical framework but emphasized different high-priority dimensions. Specifically, design solution 1 focused on trust and safety, design solution 2 prioritized ease of use and comprehensibility, and design solution 3 emphasized accessibility. Expert evaluation using the TOPSIS method revealed that design solution 2 achieved the highest overall score (C=0.877), followed by design solution 3 (C=0.728) and design solution 1 (C=0.002). These findings suggest that interfaces emphasizing clear interaction pathways, visual clarity, and immediate feedback achieve the most effective balance among trust, usability, and accessibility for older users.

### Implications for Practice and Research

In recent years, digital health technologies have played an increasingly important role in addressing loneliness and improving psychological well-being among older adults. Loneliness monitoring platforms, telehealth systems, and mobile mental health apps mainly applied wearable sensors, mobile devices, and AI-driven analytics to provide continuous monitoring and personalized interventions. These systems have shown great potential in detecting early psychosocial risks and promoting engagement in remote health care, particularly for older adults who have limited social support. However, the effectiveness of these technologies largely depends on the design of user interfaces. Therefore, ensuring older users can understand, trust, and comfortably interact with these systems is essential. Furthermore, empathic interface design that resonates with the emotional and relational dimensions of loneliness is important for fostering long-term engagement and psychological support [39]. Such designs not only convey a sense of care but also help bridge the digital divide between older adults and technology, mitigating digital loneliness through inclusive design.

Current research on loneliness and emotional well-being monitoring systems for older adults has primarily focused on usability and acceptability. For example, Choi et al [40] developed a mobile loneliness intervention app, highlighting usability, feedback clarity, and emotional support as key determinants of user acceptance. Rodríguez-Martínez et al [41] proposed a conversational chatbot designed to alleviate loneliness in older adults, emphasizing the importance of privacy and autonomy in interaction design. Xing et al [42] investigated a community engagement platform that supported older adults during the COVID-19 pandemic and underscored the role of data transparency and user control in data sharing. Additionally, Bhattacharya et al [43] demonstrated that multimodal interface design integrating voice, tactile, and visual feedback enhanced the older adults’ engagement and comprehension in telehealth applications. While these studies have identified recurring design dimensions such as usability, confidentiality, and accessibility, most relied on qualitative methods or usability testing. To address this gap, this study introduces a user-centered quantifiable hierarchical framework, shifting from single-factor evaluations to a systematic and multidimensional method for assessing interactive factors.

Theoretically, we introduced a knowledge-driven design pathway grounded in both literature insights and user requirements. Through visualized analysis and keyword co-occurrence mapping of relevant publications, we constructed a research landscape of interface design for mental health technologies in older adults. The qualitative findings were further integrated from focus group discussions, leading to the construction of an initial indicator pool. This process ensured that the theoretical foundation of the framework was both systematic and progressive, enabling the model to reflect not only the expert experience and user needs but also the academic field’s evolving focus and research trends. Methodologically, the study integrated the AHP and the TOPSIS to construct a quantifiable hierarchical framework. The AHP was applied to determine experts’ judgments on the relative importance of design dimensions, while TOPSIS was used to comprehensively evaluate and prioritize different design solutions based on these weightings, achieving a 3-level validation process spanning theory, structure, and empirical evaluation. This approach transforms interface evaluation from subjective experience into a quantifiable decision-making process, offering a novel means of representing complex psychological and emotional factors in user interface design.

To translate the quantitative results into actionable principles for digital mental health design, we highlighted some major dimensions that received high weighting in the AHP hierarchy. First, trust and safety are the most highly weighted dimensions identified through the AHP, showing that the credibility and transparency of digital platforms are essential for sustained engagement among older adults. Furthermore, significant progress has been made in strategies for health data protection and security, such as encryption technologies, anonymization protocols, and blockchain-based solutions which can improve data privacy and integrity [44-46]. These studies have shown that clear privacy principles and controllability can reduce technology-related anxiety and increase adherence to digital health systems. In the context of loneliness monitoring, fostering user autonomy not only mitigates distrust but also transforms the interaction from passive monitoring to active self-management, thereby promoting psychological empowerment among older adults [47].

Additionally, both experts and older users expressed a strong need for clarity and cognitive simplicity when interacting with digital platforms. The design solution 2 incorporated structured task flows, step-by-step prompts, and consistent layout design, which improved expert ratings for comprehensibility, feedback and support, and ease of use. These findings suggest that guided interaction models, which provide immediate feedback, linear navigation, and supportive cues, can effectively reduce cognitive load and enhance older adults’ confidence in digital self-management.

Accessibility was also identified as a key factor in age-friendly interface design. The design indicators such as offline usability under poor network conditions and accessibility support functions received high expert ratings in evaluations. However, the wireless communication technologies commonly used in smart garments and furniture such as Bluetooth and Wi-Fi still face significant challenges, particularly in terms of data quality, sampling rate, and transmission range. Data loss or latency can lead to inaccurate or incomplete monitoring, potentially resulting in misinterpretation of users’ health conditions [48]. The restricted range of Bluetooth and Wi-Fi may inadvertently reduce older adults’ mobility, especially in spacious living environments or outside the home, when precise data collection is required.

Finally, although personalization and emotional comfort received relatively lower weights in the AHP hierarchy, empathetic and adaptive design remains essential in the context of loneliness monitoring for older adults. This result can be interpreted considering the expert decision-making process. Emotional comfort may be implicitly contingent on more foundational interaction requirements, such as trust, safety, usability, and accessibility. This interpretation aligns with motivational models such as the Maslow hierarchy of needs, in which affective and self-related needs tend to be addressed only after the basic functional and safety needs are satisfied [49]. In the context of digital health interfaces, experts may therefore prioritize dimensions that reduce risk, uncertainty, and cognitive burden before considering emotional engagement. Loneliness is a subjective and context-dependent experience, influenced by emotional states, daily routines, and social relationships [50]. Therefore, interface personalization such as allowing users to adjust the tone of feedback, the frequency of reminders, or the interface mode plays a vital role in enabling the system to respond to diverse psychological and emotional needs. Moreover, emotionally supportive and adaptive design can increase older adults’ openness to using digital mental health platforms and foster a sense of emotional stability [51]. For example, emotionally supportive language can be translated into the use of positively framed feedback messages such as “You are doing well today” rather than deficit-oriented alerts [52]. In addition, concerns about psychological acceptance and perceived intrusiveness informed design decisions that minimized the salience of continuous monitoring, such as presenting sensing functions in the background and allowing users to pause data collection or customize notifications [53]. Therefore, emotional support and adaptability should not be regarded as aesthetic additions but as ethical foundations of inclusive digital health design. The goal of digital mental health technologies is not just functional usability, but to foster psychological stability and emotional connectivity through empathic interaction mechanisms tailored to the lived experiences of older adults.

The widespread adoption of digital technologies has developed new opportunities for promoting mental health among older adults while simultaneously revealing persistent digital inequalities in contemporary society. Older populations remain at a disadvantage in terms of accessing, understanding, and trusting digital systems [54,55]. This “digital divide” stems not only from differences in individual capability but also from a broader lack of inclusivity and emotional awareness in technology design. Building on 7 key dimensions, including trust and safety, comprehensibility, accessibility, and emotional comfort, this study proposes a multifactor hierarchical framework that reconceptualizes “usability” from an individual challenge to a societal design responsibility. This shift suggests that digital health systems should move beyond the narrow focus on “user experience” toward the broader pursuit of citizen well-being. As the interface serves as a mediator between people, society, and technology, it carries the social function of emotional connection and psychological support. Accordingly, the quantitative framework established in this study not only enhances the precision of digital product evaluation but also provides a theoretical pathway for achieving ethical inclusivity and social equity in digital health. Future research should further explore the application of this framework in policy evaluation, geriatric care service systems, and cross-cultural digital health contexts, promoting its integration into real-world practices that advance both technological innovation and social justice.

### Strengths and Limitations

The innovation aspect of this study lies in the development of a user-centered quantifiable prioritization framework for interface design in loneliness monitoring among older adults. We integrated qualitative focus group analysis, literature search, and expert consultation to ensure that the final indicator system was both evidence-based and grounded in user needs. Furthermore, we applied the AHP and TOPSIS to achieve the prioritization and empirical validation of interface design solutions, providing a systematic approach for future research in digital health and HCI. In addition, within the context of loneliness monitoring for older adults, psychological and emotional dimensions were integrated into the study. These dimensions are critical for engagement, empathy, and sustained interaction of older adults, but often underrepresented in existing digital health design frameworks. By combining user-centered design principles with data-driven decision analysis, this research contributes to the emerging field of digital mental health for older populations.

However, several limitations should be acknowledged. First, although the number of experts was sufficient for AHP analysis, the limited representation from gerontology and health informatics may have constrained the diversity of perspectives from psychology, data security, and clinical care. This imbalance may have influenced the weighting outcomes by placing greater emphasis on interaction efficiency and technical feasibility, while potentially underrepresenting clinically oriented considerations such as long-term care workflows, symptom interpretation, and psychosocial support needs in later life. Moreover, emotional comfort is inherently subjective and more difficult to operationalize into discrete interface features, which may lead experts to deprioritize it relative to more tangible and measurable design dimensions within a hierarchical decision-making process. Future research should expand the expert pool to enhance representativeness and better capture affective and experiential priorities. Second, the evaluation of design solutions in this study mainly relied on expert judgment and expert-based eye-tracking assessment rather than real-world usability testing with older adults. While the use of experts was appropriate for early-stage evaluation of information architecture, expert participants may not fully represent the emotional characteristics of older adults. Future studies can validate these findings through user trials and incorporate user feedback into the iterative design process. As the user interface developed in this study was built upon our smart textile loneliness monitoring system (the DELONELINESS system), further research is required to examine the usability, accessibility, and user acceptability of the integrated system through follow-up usability testing and in-situ evaluation with older adults to verify whether expert-informed design priorities align with real-world user experiences. Finally, future work may explore the integration of this framework within broader health policy contexts, such as the United Kingdom’s National Health Service digital care strategies or other international systems, to adapt the model to evolving user needs and technological ecosystems.

### Conclusions

This study developed a user-centered quantifiable prioritization framework for interface design in loneliness monitoring among older adults. By integrating qualitative insights from focus groups, literature-derived indicators, and expert consultation, the study established a comprehensive design indicator system based on the AHP and evaluated design solutions using the TOPSIS. Using user-centered design principles with data-driven decision analysis, this research not only provides a systematic design framework for future research in HCI, but also bridges subjective user experience with objective design evaluation, offering practical insights for developing empathic and inclusive digital mental health technologies for older adults. Beyond the DELONELINESS system, the proposed framework can be adapted to inform the design and evaluation of other digital mental health platforms and age-friendly interactive systems by supporting structured prioritization of usability, trust, accessibility, and emotional considerations across diverse contexts.

## Supplementary material

10.2196/88263 Multimedia Appendix 1 Qualitative thematic analysis of focus groups.

10.2196/88263 Multimedia Appendix 2 Analytic hierarchy process questionnaire.

10.2196/88263 Multimedia Appendix 3 Weights of indicators for the DELONELINESS (Design for Healthy Ageing: a Smart System to Decrease Loneliness for Older People) system interface design.

## Abbreviations

AHP: analytic hierarchy process
AI: artificial intelligence
CR: consistency ratio
DELONELINESS: Design for Healthy Ageing: a Smart System to Decrease Loneliness for Older People
HCI: human-computer interaction
ICC: intraclass correlation coefficient
TCT: task completion time
TOPSIS: Technique for Order Preference by Similarity to Ideal Solution
