# Supplementary material for: A User-Centered Interface Design Framework for the DELONELINESS System in Older Adults: Design Indicator Development and Prioritization
Source: JMIR Hum Factors. 2026 Mar 6;13:e88263. doi: 10.2196/88263 (PMC12978934; doi:10.2196/88263)
Supplement: Multimedia Appendix 1 [file humanfactors-v13-e88263-s001.docx]

| **Theme** | **Example Quote** | **Interface Design Indicator** |
| --- | --- | --- |
| Comprehensibility | *“I wouldn’t want lots of numbers or graphs... just a clear sign if something is wrong.”*  *“Sometimes it’s hard to tell what all the icons mean. Maybe just use words.”* | Clarity of visual elements |
|  | *“If you explain too much, I won’t read it. Just tell me what matters.”*  *“Sometimes I wouldn’t want to scroll through menus. Just show me what I need on the home screen.”* | Simplicity of information |
|  | *“I might need someone to show me first or maybe a short video to explain it.”* | Provision of tutorial/demo mode |
| Trust and Safety | *“I want to know what it’s collecting, not just get a result without knowing why.”*  *“What exactly is it tracking? I’d feel better if I could see that.”* | Transparent data collection |
|  | *“I don’t want it to start suggesting things unless I’ve turned that on myself.”*  *“I’d like a clinician to review the data before contacting me.”* | User authorization of functions |
|  | *“Can I decide who sees what? Like family yes, GP maybe not.”*  *“Sometimes I just want to turn bits off, like the reminders or the emotional check-in.”* | User ability to modify permissions |
| Feedback and Support | *“I’d like it to show me how I’ve been feeling recently, maybe a summary chart or something visual.”* | Real-time health status feedback |
|  | *“If I get a notification saying I’m lonely, I want to know what to do next. Like, what are my options?”*  *“Could it offer suggestions automatically? Like ‘maybe go out for a walk or call someone’ kind of thing?”*  *“I just want to clearly see what to do next.”* | Immediate guidance |
|  | *“If I’m confused, I’d like to be able to ask someone straight from the app.”* | Online assistance |
| Emotional Comfort | *“I wouldn’t want it to tell me something’s wrong in a way that makes me panic.”*  *“Too much information may also makes me anxious.”* | Emotionally supportive language |
|  | *“A picture of someone smiling or drinking tea can help, it’s not all about numbers.”* | Emotionally supportive image |
|  | *“If it knows I usually read in the evening, maybe it could check in gently at that time.”* | Integration with daily life routines |
| Personalization | *“Can the text be a bit bigger? My eyesight isn’t great these days.”* | Customizable fonts |
|  | *I’d prefer the app used simpler terms ‚ I don’t want it feels too clinical.”*  *“The UK is a diverse country. Is this app only for English speakers?“* | Customizable language |
|  | *“I‘d like to turn off some reminders, or choose when they come through.”* | Customizable notifications |
|  | *“Sometimes I don’t want a complicated dashboard, just a simple daily check-in.”*  *“Can I access this APP without WIFI?”* | Customizable mode |
|  | *“I hope I can modify my emotional state, as loneliness is a very subjective emotion and the system might make mistakes.”* | Customizable mood input |
