# Supplementary material for: A User-Centered Interface Design Framework for the DELONELINESS System in Older Adults: Design Indicator Development and Prioritization
Source: JMIR Hum Factors. 2026 Mar 6;13:e88263. doi: 10.2196/88263 (PMC12978934; doi:10.2196/88263)
Supplement: Multimedia Appendix 2 [file humanfactors-v13-e88263-s002.docx]

**Analytic Hierarchy Process (AHP) Questionnaire**

**Instructions:**

Please compare the relative importance of the following indicators.

If the indicator in the row (horizontal A) is more important than the indicator in the column (vertical B), select an integer (1, 2, 3, 4, 5, 6, 7, 8, 9). The larger the number, the more important A is compared to B.

If the indicator in the column (vertical B) is more important than the indicator in the row (horizontal A), select the reciprocal (1/2, 1/3, 1/4, 1/5, 1/6, 1/7, 1/8, 1/9). The larger the denominator, the more important B is compared to A.

**1. Level 1 Indicators**

|  | C1 | C2 | C3 | C4 | C5 | C6 | C7 |
| --- | --- | --- | --- | --- | --- | --- | --- |
| C1  Comprehensibility | \ | \ | \ | \ | \ | \ | \ |
| C2  Ease of Use |  | \ | \ | \ | \ | \ | \ |
| C3  Trust & Safety |  |  | \ | \ | \ | \ | \ |
| C4  Feedback & Support |  |  |  | \ | \ | \ | \ |
| C5  Emotional Comfort |  |  |  |  | \ | \ | \ |
| C6  Personalization |  |  |  |  |  | \ | \ |
| C7  Accessibility |  |  |  |  |  |  | \ |

(If both indicators are equally important, select 1.)

**2. Level 2 Indicators (C1-Comprehensibility)**

|  | C1-1 | C1-2 | C1-3 | C1-4 |
| --- | --- | --- | --- | --- |
| C1-1  Consistency of interface layout | \ | \ | \ | \ |
| C1-2  Clarity of visual elements |  | \ | \ | \ |
| C1-3  Simplicity of information content |  |  | \ | \ |
| C1-4  Provision of tutorial/demo mode |  |  |  | \ |

**3. Level 2 Indicators (C2 – Ease of Use)**

|  | C2-1 | C2-2 | C2-3 | C2-4 |
| --- | --- | --- | --- | --- |
| C2-1  Clear interaction areas | \ | \ | \ | \ |
| C2-2  Support for multimodal interaction |  | \ | \ | \ |
| C2-3  Easy correction of errors |  |  | \ | \ |
| C2-4  Clear interaction pathways |  |  |  | \ |

**4. Level 2 Indicators (C3 – Trust and Safety)**

|  | C3-1 | C3-2 | C3-3 |
| --- | --- | --- | --- |
| C3-1  Transparent data collection | \ | \ | \ |
| C3-2  User authorization of functions |  | \ | \ |
| C3-3  User ability to modify permissions |  |  | \ |

**5. Level 2 Indicators (C4 – Feedback & Support)**

|  | C4-1 | C4-2 | C4-3 |
| --- | --- | --- | --- |
| C4-1  Real-time health status feedback | \ | \ | \ |
| C4-2  Immediate guidance |  | \ | \ |
| C4-3  Online assistance |  |  | \ |

**6. Level 2 Indicators (C5 – Emotional Comfort)**

|  | C5-1 | C5-2 | C5-3 |
| --- | --- | --- | --- |
| C5-1  Emotionally supportive language | \ | \ | \ |
| C5-2  Emotionally supportive images |  | \ | \ |
| C5-3  Integration with daily life scenarios |  |  | \ |

**7. Level 2 Indicators (C6 – Personalization)**

|  | C6-1 | C6-2 | C6-3 | C6-4 | C6-5 |
| --- | --- | --- | --- | --- | --- |
| C6-1  Customizable fonts | \ | \ | \ | \ | \ |
| C6-2  Customizable language |  | \ | \ | \ | \ |
| C6-3  Customizable notifications |  |  | \ | \ | \ |
| C6-4  Customizable mode |  |  |  | \ | \ |
| C6-5  Customizable mood input |  |  |  |  | \ |

**8. Level 2 Indicators (C7 – Accessibility)**

|  | C7-1 | C7-2 | C7-3 | C7-4 |
| --- | --- | --- | --- | --- |
| C7-1  Cross-device access | \ | \ | \ | \ |
| C7-2  Multi-system compatibility (iOS/Android, etc.) |  | \ | \ | \ |
| C7-3  Accessibility support functions (screen reader, captions, magnifier, etc.) |  |  | \ | \ |
| C7-4  Offline usability under poor network conditions |  |  |  | \ |
