# Supplementary material for: A User-Centered Interface Design Framework for the DELONELINESS System in Older Adults: Design Indicator Development and Prioritization
Source: JMIR Hum Factors. 2026 Mar 6;13:e88263. doi: 10.2196/88263 (PMC12978934; doi:10.2196/88263)
Supplement: Multimedia Appendix 3 [file humanfactors-v13-e88263-s003.docx]

**Weights of indicators for the DELONELINESS system interface design**

| First-level indicators | Weights | Second-level indicators | Weights |
| --- | --- | --- | --- |
| 1. Comprehensibility | 0.157 | 1.1 Consistency of interface layout | 0.037 |
|  |  | 1.2 Clarity of visual elements | 0.039 |
|  |  | 1.3 Simplicity of information content | 0.039 |
|  |  | 1.4 Provision of tutorial/demo mode | 0.043 |
| 2. Ease of Use | 0.187 | 2.1 Clear interaction areas | 0.052 |
|  |  | 2.2 Support for multimodal interaction | 0.029 |
|  |  | 2.3 Easy correction of errors | 0.039 |
|  |  | 2.4 Clear interaction pathways | 0.067 |
| 3. Trust and Safety | 0.206 | 3.1 Transparent data collection | 0.057 |
|  |  | 3.2 User authorization of functions | 0.066 |
|  |  | 3.3 User ability to modify permissions | 0.084 |
| 4. Feedback and Support | 0.111 | 4.1 Real-time health status feedback | 0.047 |
|  |  | 4.2 Immediate guidance | 0.038 |
|  |  | 4.3 Online assistance | 0.026 |
| 5. Emotional Comfort | 0.080 | 5.1 Emotionally supportive language | 0.022 |
|  |  | 5.2 Emotionally supportive images | 0.023 |
|  |  | 5.3 Integration with daily life scenarios | 0.035 |
| 6. Personalization | 0.092 | 6.1 Customizable fonts | 0.015 |
|  |  | 6.2 Customizable language | 0.033 |
|  |  | 6.3 Customizable notifications | 0.020 |
|  |  | 6.4 Customizable mode | 0.010 |
|  |  | 6.5 Customizable mood input | 0.013 |
| 7. Accessibility | 0.167 | 7.1 Cross-device access | 0.029 |
|  |  | 7.2 Multi-system compatibility (iOS/Android, etc.) | 0.039 |
|  |  | 7.3 Accessibility support functions (screen reader, captions, magnifier, etc.) | 0.044 |
|  |  | 7.4 Offline usability under poor network conditions | 0.055 |
